# A180 THE IMPACT OF INTEGRATING PELVIC MRI AT DIAGNOSIS FOR EARLY DETECTION OF PERIANAL CROHN’S DISEASE IN PEDIATRICS

**DOI:** 10.1093/jcag/gwae059.180

**Published:** 2025-02-10

**Authors:** M Antaya, A Hudson, E Lerner, K Nasser, M Carroll, D Migliarese Isaac, E Wine, T Perry, A Thompson, H Huynh

**Affiliations:** Pediatrics, University of Alberta Faculty of Medicine & Dentistry, Edmonton, AB, Canada; Division of Pediatric Gastroenterology, Department of Pediatrics, University of Alberta, Edmonton, AB, Canada; Division of Pediatric Surgery, Department of Surgery, University of Alberta, Edmonton, AB, Canada; Division of Pediatric Surgery, Department of Surgery, University of Alberta, Edmonton, AB, Canada; Division of Pediatric Gastroenterology, Department of Pediatrics, University of Alberta, Edmonton, AB, Canada; Division of Pediatric Gastroenterology, Department of Pediatrics, University of Alberta, Edmonton, AB, Canada; Division of Pediatric Gastroenterology, Department of Pediatrics, University of Alberta, Edmonton, AB, Canada; Division of Pediatric Surgery, Department of Surgery, University of Alberta, Edmonton, AB, Canada; Division of Pediatric Radiology, Department of Radiology & Diagnostic Imaging, University of Alberta, Edmonton, AB, Canada; Division of Pediatric Gastroenterology, Department of Pediatrics, University of Alberta, Edmonton, AB, Canada

## Abstract

**Background:**

Perianal Crohn’s disease (CD) can be a severe, refractory manifestation of pediatric CD. It is underrecognized, particularly if asymptomatic, which has led to varied reported incidences (10-60%). Earlier detection by pelvic magnetic resonance imaging (pMR) may alter management and outcomes.

**Aims:**

If performing pMR on newly diagnosed pediatric CD patients identifies asymptomatic perianal CD and leads to earlier biologic use, fewer hospitalizations, and less surgery.

**Methods:**

New CD patients were prospectively enrolled into the Edmonton Pediatric Inflammatory Bowel Disease Clinic (EPIC) registry. This clinic has done baseline pMR since 2018. A retrospective review (2018-2023) collected pMR, perianal exam, symptoms, blood work, calprotectin, medications, and surgeries (perianal and intestinal). A blinded radiologist re-read pMRs using St. James and Parks criteria. Statistics included descriptive, Fisher’s exact test, multivariate logistic regression, and survival analysis.

**Results:**

139 patients were included (63% male, median age 13 [IQR 11-16]). Ninety-six patients (69%) had no pMR perianal disease (pMR-), despite 13/96 (14%) having perianal symptoms and 26/96 (27%) with inflamed (n=6)/ non-inflamed (n=14) tags or fissures (n=6). Of the 43 (31%) with pMR+ perianal disease, 27/43 (63%) were asymptomatic (ASx) and 17/43 (40%) had a normal exam. pMR+/ASx patients with perianal exam findings included inflamed (n=4)/ non-inflamed (n=5) tags, fissures (n=1), and draining fistula (n=1). pMR+/Sx had higher Parks and St James scores compared to pMR+/ASx. Compared to pMR-, pMR+/ASx and pMR+/Sx patients had a relative risk of 1.40 [95% CI 1.16-1.71] and 1.72 [95% CI 1.72-1.73] of requiring a biologic at 6 months, and a relative risk of 1.39 [95% CI 1.23-1.62] and 1.45 [95% CI 1.45-1.46] of requiring a biologic at 1 year. pMR+/Sx patients had an increased probability of CD surgery (p<0.002) but not hospitalization (p>0.05). There were no differences between pMR+/ASx and pMR- patients in surgery or hospitalization (p>0.05). Time to surgery was shorter in pMR+/Sx (p<0.01), but not different between pMR+/ASx and pMR- (p>0.05).

**Conclusions:**

The majority of patients with perianal CD identified on pMR had no perianal symptoms and had a normal exam. This early identification of subclinical perianal disease led to increased and earlier biologic use, which may explain the lack of increased hospitalization and surgical rates compared to those without pMR+ perianal disease. Baseline imaging was also helpful for demonstrating the absence of deeper perianal disease in several patients who did have perianal symptoms and tags/fissures.

**Funding Agencies:**

NACTRC, Stollery Children’s Hospital Foundation through WCHRI

